# Glioblastoma vaccine tumor therapy research progress

**DOI:** 10.1186/s41016-021-00269-7

**Published:** 2022-01-19

**Authors:** Tong Zhao, Chunwang Li, Hongliang Ge, Yuanxiang Lin, Dezhi Kang

**Affiliations:** 1 Department of Neurosurgery, Neurosurgery Research Institute, the First Affiliated Hospital, Fujian Medical University, Fuzhou, Fujian Province People’s Republic of China; 2 Fujian Key Laboratory of Precision Medicine for Cancer, the First Affiliated Hospital, Fujian Medical University, Fuzhou, Fujian Province People’s Republic of China; 3 Key Laboratory of Radiation Biology of Fujian higher education institutions, the First Affiliated Hospital, Fujian Medical University, Fuzhou, Fujian Province People’s Republic of China; 4 Clinical Research and Translation Center, the First Affiliated Hospital, Fujian Medical University, Fuzhou, Fujian Province People’s Republic of China; 5 Fujian Institute for brain disorders and brain science, the First Affiliated Hospital, Fujian Medical University, Fuzhou, Fujian Province People’s Republic of China

**Keywords:** Glioblastoma, Vaccine therapy, Immunotherapy, Tumor microenvironment, Review

## Abstract

Glioblastoma (GBM) is the most common primary malignancy of the central nervous system in adults. The prognosis for late-stage glioblastoma (World Health Organization grade IV astrocytic glioma) is very poor. Novel treatment options are sought after and evaluated by clinicians and researchers, and remarkable advances have been made in surgical techniques, radiotherapy, and chemotherapy. However, the treatment of glioblastoma remains extremely difficult and it can extend the lives of patients by only a few months. There has been notable progress in the field of immunotherapy, particularly with the use of tumor vaccines, for treating glioblastoma; especially peptide vaccines and cell-based vaccines such as dendritic cell vaccines and tumor cell vaccines. However, the results of the current clinical trials for vaccination are not satisfactory. This article reviews the progress in the development of vaccines for glioblastoma.

## Background

Glioblastoma (GBM) is a primary central nervous system cancer with an annual incidence of approximately 3.19/100, 000, very poor prognosis, and a very short median survival time of approximately 14.6–16.6 months [1–4]. GBM is primarily treated through surgery to clear the pathological features, followed by radiotherapy and temozolomide azole synchronization amine chemotherapy, and finally, adjuvant chemotherapy. Limitations such as the heterogeneity of the tumor, the blood-brain barrier, and the immunosuppression of gliomas affect the efficacy of existing treatment regimens [5, 6]. Novel treatment strategies are explored and some progress has been made, such as in tumor immunotherapy, especially in vaccine therapy. Vaccine therapy is based on the tumor-specific immune response to the injected exogenous antigens. The introduction of foreign antigens to antigen-presenting cells induces and enhances the immunity of the host. The current clinical trials on vaccines for GBM are primarily peptide-based vaccines and cells of Phytophthora seedlings. This paper reviews the promising strategies of vaccine therapies for treating GBM.

### Peptide vaccines

GBM is characterized by a large number of mutations; however, GBM due to mutation has a relatively low negative charge notorious [7]. The protein/peptide variants encoded from the mutated gene are unique to the tumor cells and not present in normal cells; therefore, they can be used as specific antigens for eliciting immune responses against tumor cells. These antigens are referred to as tumor-specific antigens (TSAs), earlier described as “neo-antigens”. Only a few mutations are processed into new epitopes; when presented by the antigen presenting cells in the human leukocyte antigen (human leukocyte the antigen, HLA) on presentation, these epitopes result in T cell-based immunity. Many potential tumor antigens do not originate from mutations, but from erroneous or overexpression of normal proteins that are also expressed in other tissues. In such cases, targeting the antigen may lead to autoimmunity, resulting in non-target effects such as brain inflammation [8]. The lack of specificity and high expression of epitopes in GBM are limiting factors in the development of peptide vaccine-based strategies.

### Epidermal growth factor receptor type III mutant

Epidermal growth factor receptor III type mutant (Eggroll) remains the most relevant and undisputed TSA in GBM, found in 20–30% of the tumors. In the late 1990s, researchers designed a peptide vaccine against a TSA (CDX-110), to recognize and promote immune responses against the mutant sequences. CDX-110 has a good preclinical efficacy in mouse brain tumor models, in terms of inducing humoral and cytotoxic T cell responses [9]. Based on the results from early clinical data [10], a multi-center, dual-arm phase III clinical trial (ACT IV) was conducted, with 745 newly diagnosed GBM patients. Patients receiving rindopepimut exhibited a good humoral immune response, compared to the patient’s in the control group; however the median overall survival did not improve significantly [11]. Abnormally low cut-off values that are positive for Egeria affect the test results. In addition, researchers have developed a drug that targets Egeria (ADU-623) and a phase I clinical trial in patients with recurrent high-grade glioma, was conducted. However, the disappointing results from the clinical trial of a CTIV significantly slowed the development of the Egeria-targeting peptide vaccine.

### Isocitrate dehydrogenase 1

Mutation in isocitrate dehydrogenase (IDH) does not occur in normal human cells; it occurs almost exclusively in tumor cells, making it a promising TSA [12]. Approximately 80% of low-grade gliomas have IDH mutations; among them, the R132H mutation in IDH1 rarely occurs in primary GBM. The presence of an IDH1 gene mutation indicates that the GBM is a secondary low-grade glioma. Peptides targeting R132H induce antigen-specific CD4+ T cells and humoral responses, following the appearance of MHC class II (lack of class I epitopes) [13]. Currently, phase I clinical trials of peptide vaccines targeting IDH1 R132H are ongoing.

### Peptide vaccine

The mutation load of GBM is relatively low [7]; however, tumor heterogeneity remains an obstacle, especially for selective single-target therapy. Such treatment can be limited by antigen escape, where the tumor no longer expresses the target antigen [14]. Therefore, it is essential to develop a model that identifies and combines multiple novel antigens and predicts HLA presentation capabilities, which is a question of priorities. Two recently published key trials have highlighted the trend of personalized cancer vaccines against novel antigens [15, 16]. In the first study, a personalized cancer vaccine was developed against a novel antigen, identified through comparing the whole exon sequence data from the resected tumor and the matched normal tissues [16]. For each patient, 7 to 20 antigens that were predicted to have a high affinity for HLA type-I binding were chosen for vaccine development. The second study combined two novel antigens and non-mutated tumor-associated antigens to increase the number of binding epitopes [15]. Nine by the non-mutated peptides (APVAC1 patient) to a vaccine composition after injection, followed by the administration of 20 peptides of new antigens (APVAC 2). Both studies were phase I clinical trials; they could induce a considerable number of invasive tumor-reactive T memory cells and clonal expansion of antigen-specific cells.

### Cell-based vaccines

There are two main cell-based antitumor vaccines: the tumor cell vaccine and dendritic cell (DC) vaccine.

#### Dendritic cell vaccine

The treatment of GBM vaccine clinical trials currently under way is the most clinically available DC vaccine clinical trial. DCs are the strongest antigen-presenting cells in humans; they induce innate immunity, acquired immunity, and enable immunity conversion. In addition, they also influence the immune responses of lymphocytes, differentiation, and antigen presentation [17]. DCs were discovered by Steinman in 1973; however, its key role in the immune response were established only in the early 1990s [18]. DC vaccine preparation and inoculation involves isolating the DCs from the patient, loading them with tumor antigens and treating them with the corresponding cytokines to induce maturity, and finally the preparation of human DC vaccines for re-injection into the patient [19]. This DC vaccine preparation process is a reasonable anti-tumor vaccine strategy, majorly because it formed the main body of silence-T; it is the first FDA-approved cancer vaccine. Sepulture-T is demonstrated to be clinically efficient in improving the median overall survival period in prostate cancer patients of 4 months [20]. For treating GBM with DC vaccine, DCs are isolated from the peripheral blood CD-14 positive monocytes and GM-CSF and IL-4 are used to induce the differentiation of immature DCs [21]. The tumor antigens (including polypeptide, RNA, DNA, and tumor lysates) are loaded into the immature DCs, which are then presented on MHCs, and the various cytokines (for example, of GM-CSF, of IL-.4, of TNF-α, and IL-6 under) action to maturity [21, 22]. The USA, Europe, and Japan have published a lot of respect for the use of DC vaccine therapy in glioma clinical research paper [23]. The Department of Neurosurgery in our hospital is also actively conducting a phase II clinical trial of a DC vaccine and found that the DC vaccine marginally improves the survival period of GBM patients [24]. However, there is still no clear evidence for testing the efficacy in a phase III clinical trial, and the production of vaccines is very expensive. Diva is a DC vaccine project developed by Northwest Biotherapeutics based on the research of Linda Lieu et al. [25]; it is presently in phase III clinical trials. The latest developments in DC vaccines include the pretreatment of vaccine sites. Dendritic cells carrying cytomegalovirus phosphoprotein 65 (pp65) RNA significantly improve lymph node homing and prolong the overall survival time, following the pretreatment of the vaccine site with tetanus/diphtheria antigens [26].

#### Tumor cell vaccine

Early vaccines often used killed or inactivated tumor cells, similar to that of antiviral vaccines. The success rate was relatively low; and therefore gene-editing tumor cells were initiated in the late 1980s, for expressing some immune-stimulating cytokines; granulocyte macrophage colony-stimulating factor (GM-CSF) was most commonly used. Tumor cells secreting GM-CSF are evaluated for treating GBM [27]. Phase I clinical trials are completed using the latest generation of autologous tumor cells and allogeneic tumor cell lines secreting GM-CSF (K-562). The success of vaccination is influenced by the activation of T cells and anti-tumor immunity [28]. In addition, direct injection of formalin-fixed GBM as an antigen in treating GBM, is explored [29, 30]. The overall survival in a clinical trial evaluating DC vaccines in 24 GBM patients was 22.2 months [30].

## Discussion

GBM is the most common type of brain tumor and deservedly one of the deadliest cancers. The average survival period is only 1 year and the 5-year survival rate is approximately 5% [31]. At present, maximal safe resection followed by CCRT and adjuvant chemotherapy with temozolomide is regarded as the standard treatment in GBM [32]. However, it can prolong significantly the patients’ survival, rather than thoroughly cure GBM. In addition, GBM infiltrates the brain in its early stages and spreads quickly, with distant metastases occurring later, making it difficult to completely cure GBM by a single means. In addition, the blood-brain barrier is an effective barrier against bacterial-viral invasion of the brain, but it can also make it difficult or impossible for many therapeutic agents to reach the brain. As a result, no new drug has been approved for nearly three decades, and the only available drug for brain tumors is temozolomide. Even more unfortunately, glioblastoma has a high chance of recurrence [33], and once it does, treatment options are very limited and not very effective. From surgery, to modern chemotherapy, radiotherapy, targeted therapy, our means of fighting against the disease are constantly advancing. In recent years, immunotherapy for tumors has gradually become a hot topic, among which, vaccine therapy provides a new treatment option for GBM patients, and there are numerous reports on the correlation between immune response and clinical outcome of cancer patients after receiving vaccine therapy. The “tumor vaccine” has already achieved initial results in immunotherapy. The key to vaccine therapy is the selection of the target of immunotherapy, and also, how to reduce the toxicity of the vaccine is an issue that needs close attention. The improvement of vaccines for GBM and other cancers should be based on the maximum potential of anti-tumor immune cells, the local tolerance of the tumor to immune factors, and the minimization of potential side effects. To date, most GBM vaccines have used tumor lysates without characteristic autologous homologs as antigens. This requires that the method of vaccine preparation be tailored to the individual and that the risks arising from the inclusion of normal brain tissue components in the vaccine be assumed. Such vaccines may therefore induce severe, injurious immune responses against normal brain tissue in some animal models. To date, however, there have been no serious side effects caused by tumor lysates. Typical side effects from such immunization treatments include hypothermia, rash, and pain at the vaccination site. To avoid damage to normal tissues of the human brain, several research groups have turned to vaccine development with tumor-specific antigens.

## Conclusion

The progress in developing vaccines for treating GBM is still limited. Limitations in the access to the central nervous system and the tumor due to restrictions on the choice of drug and route of immunization, heterogeneity of the tumors, low mutation negative charge, pose unique challenges. The current results from the clinical trials on vaccines for GBM are not very promising; however, with further optimization, they could develop into a unique therapeutic strategy with great potential. The investment in the research on vaccines for treating GBM needs to be improved.

## Data Availability

Not applicable

## Abbreviations

GBM: Glioblastoma
TSAs: Tumor-specific antigens
HLA: Human leukocyte the antigen
IDH: Isocitrate dehydrogenase
DC: Dendritic cell
